# Erythema Elevatum Diutinum: A Rare Case Wth Atypical Presentation

**DOI:** 10.7759/cureus.82719

**Published:** 2025-04-21

**Authors:** Karthika Ps, Manimaran R, Koppolu Kanchana, Srinivasan C, Ankita Swarnkar

**Affiliations:** 1 General Surgery, Shree Balaji Medical College and Hospital, Chennai, IND; 2 Plastic Surgery, Shree Balaji Medical College and Hospital, Chennai, IND

**Keywords:** arthritis, chronic vascular dermatosis, erythema elevatum diutunum, myositis, painful lesion

## Abstract

Erythema elevatum diutinum (EED) is an uncommon, chronic vascular dermatosis characterized by plaques, nodules, and violaceous papules, typically affecting the extensor regions of the arms and legs. Although the etiology remains unclear, associations with autoimmune disorders, infections, and malignancies have been proposed. This case report presents a 56-year-old woman, non-diabetic, with an atypical presentation of EED, featuring painful lesions in contrast to the usual painless manifestations. The patient initially presented with painless nodules two years prior, which later became painful, involving multiple sites such as the hands, feet, knees, and back. Diagnostic evaluations, including histopathological examination, confirmed EED. The atypical pain prompted further investigations, ruling out underlying systemic conditions like arthritis or myositis. The treatment consisted of surgically removing the lesions and applying split-thickness skin grafts (SSG) to alleviate persistent symptoms and enhance quality of life. The case underscores the diagnostic challenges posed by atypical EED presentations, such as pain, and highlights the importance of a multimodal approach, including surgical intervention when medical therapy proves insufficient. The purpose of this report is to highlight the range of clinical manifestations in EED and the necessity of personalized treatment strategies to manage unusual symptoms effectively.

## Introduction

Erythema elevatum diutinum (EED) is a rare, chronic form of leukocytoclastic vasculitis [1]. This distinctive inflammatory condition presents as persistent violaceous to red brown papular, plaque-like, and nodular lesions, frequently found on extensor surfaces. The symmetric involvement of hands, elbows, knees, and ankles represents the classic presentation, though clinical variations do occur. While epidemiologic studies demonstrate equal gender distribution, emerging evidence suggests a modest male predominance, particularly in patients aged 30-60 years [2]. The pathophysiology of EED remains incompletely understood, though current evidence points to immune complex-mediated vasculitis as the primary mechanism. Histopathologic examination usually shows abundant neutrophils with fibrinoid degeneration of vascular walls [3]. Clinicians should maintain a high index of suspicion for associated systemic conditions, as EED frequently coexists with autoimmune disorders (particularly rheumatoid arthritis and systemic lupus erythematosus, SLE), hematologic abnormalities (notably immunoglobulin (Ig)A paraproteinemia), and chronic infections including HIV and streptococcal pharyngitis [4-6]. Dapsone remains the cornerstone of therapy, exerting its therapeutic effect through neutrophil inhibition [7]. Alternative regimens incorporating colchicine, systemic corticosteroids, or immunomodulators may be considered in refractory cases [8].

The diagnostic challenge intensifies when encountering atypical presentations, whether in morphology, distribution, or symptomatology, as these may closely mimic other inflammatory or rheumatologic conditions [9]. This case presentation underscores the importance of comprehensive evaluation, including histopathologic confirmation, when faced with unusual manifestations of EED. The present case illustrates these diagnostic complexities while highlighting key management considerations for this uncommon entity.

## Case presentation

**Figure 1:**
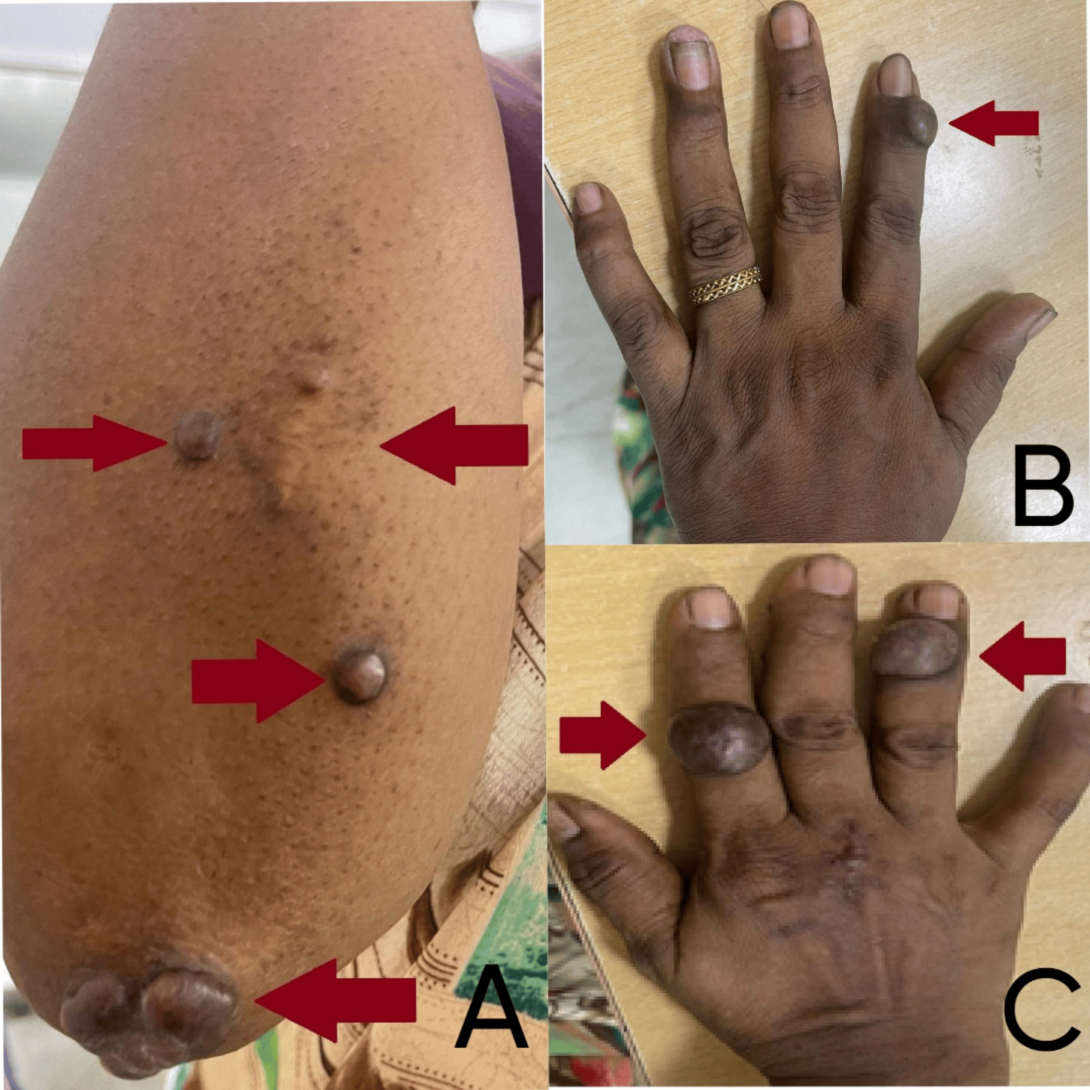
Pre-operative images of the patient A. 2 x 2 cm multiple painful nodules on the elbow region, B. 2 x 2 cm painful nodule in the anterior aspect of left index finger, C. 2 x 2 cm painful nodule in the anterior aspect of right index and ring finger.

A 56-year-old female patient presented with multiple painful swellings for the past one week involving the back and extremities, which were painless two years back when the patient was presented initially. Routine blood tests, inflammatory markers, erythrocyte sedimentation rate, C-reactive protein, basic metabolic panel, and autoimmune workup were normal when checked initially. Histopathological examination of the skin biopsy confirmed the diagnosis of EED two years ago. On examination, multiple tender, soft swellings, each measuring 2 x 2 cm nodules, were found at the posterior and medial aspect of the right elbow, left hand, and right hand (Figure 1), the medial and lateral aspect of the right foot, and the dorsal and plantar aspect of the left foot. The lesions were hyper- and hypopigmented with intact sensation and a normal range of motion in the joints involved.

**Figure 2:**
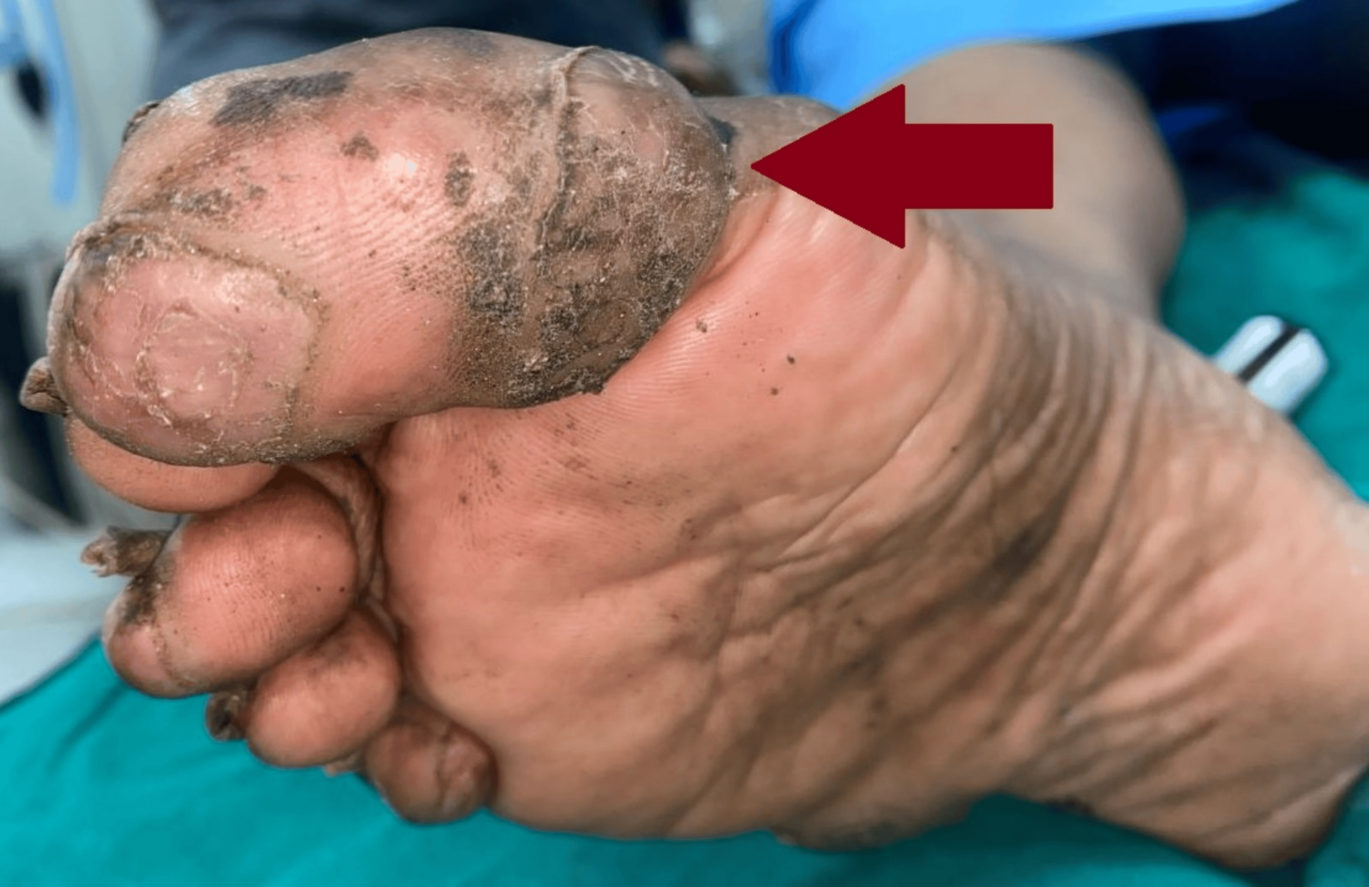
Pre-operative image of the patient's right foot 2 x 2 cm multiple painful nodules on the lateral part of the right foot.

**Figure 3:**
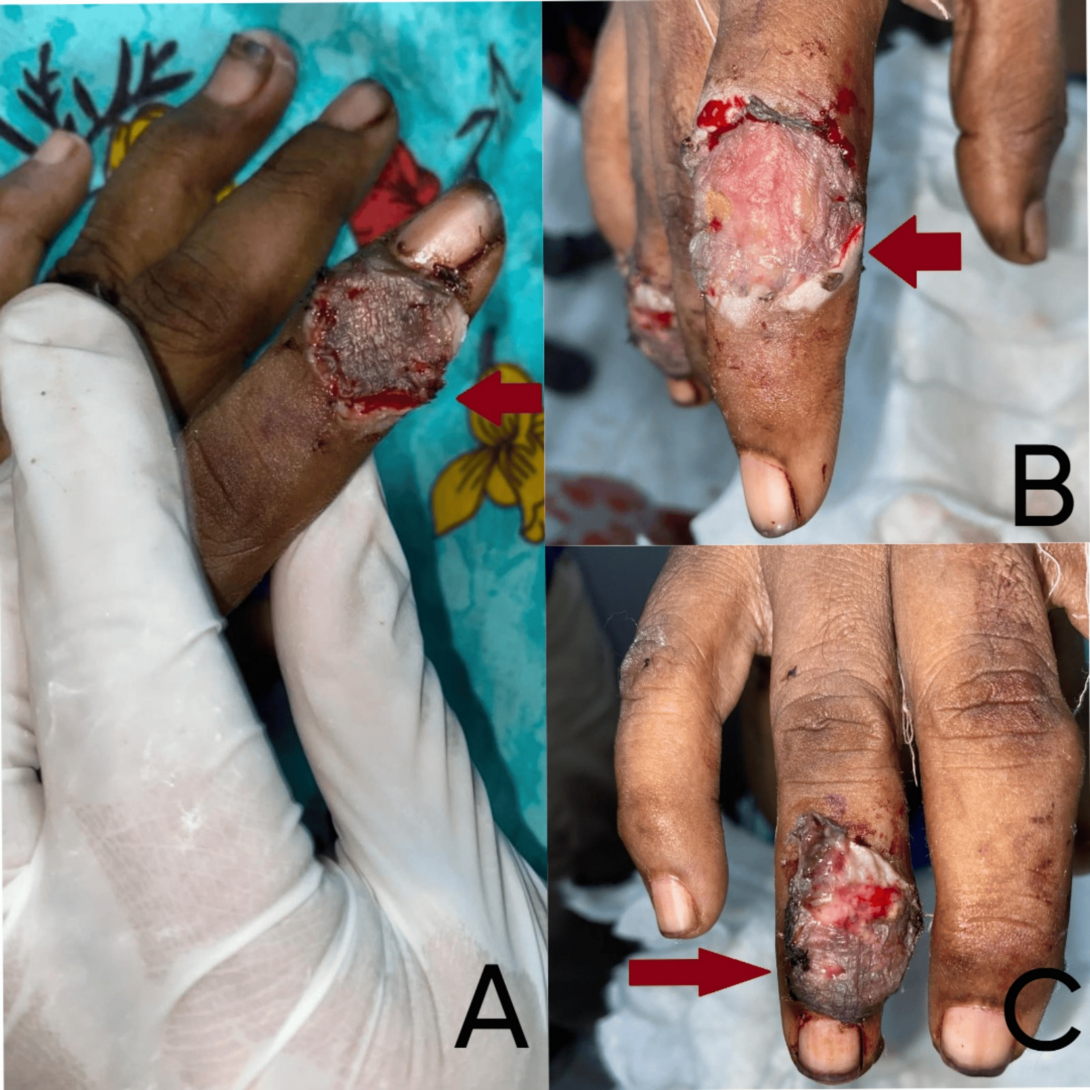
Post-operative images of the patient A. Excision with split-thickness skin grafts (SSG) over left index finger, B. Excision with SSG over right index finger, C. Excision with SSG over right ring finger.

**Figure 4:**
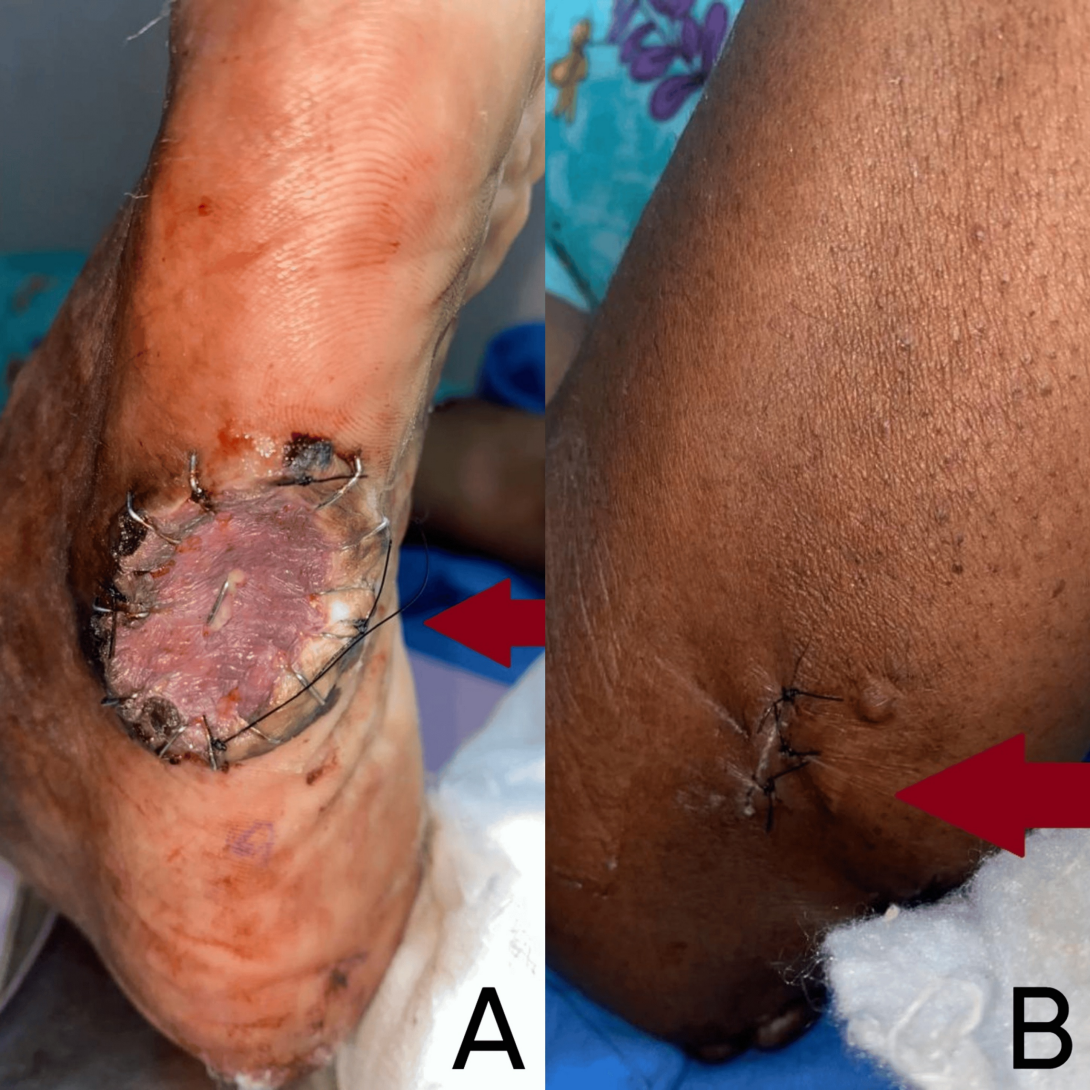
Post-operative image of the patients limbs A. Excision with split-thickness skin grafts (SSG) over the right foot, B. Excision with SSG over the elbow region.

Anesthesia fitness was obtained. Under aseptic preparation and general anesthesia in supine position, the affected parts were painted and draped. Elliptical incisions were made around the lesion. The incisions were deepened and lesions were excised for biopsy. A split-thickness skin graft was then harvested from thigh and meticulously tailored to fit the raw areas (Figures 3-4). The SSG graft was then placed and secured over the recipient sites. Following surgery, the patient improved well.

**Figure 5:**
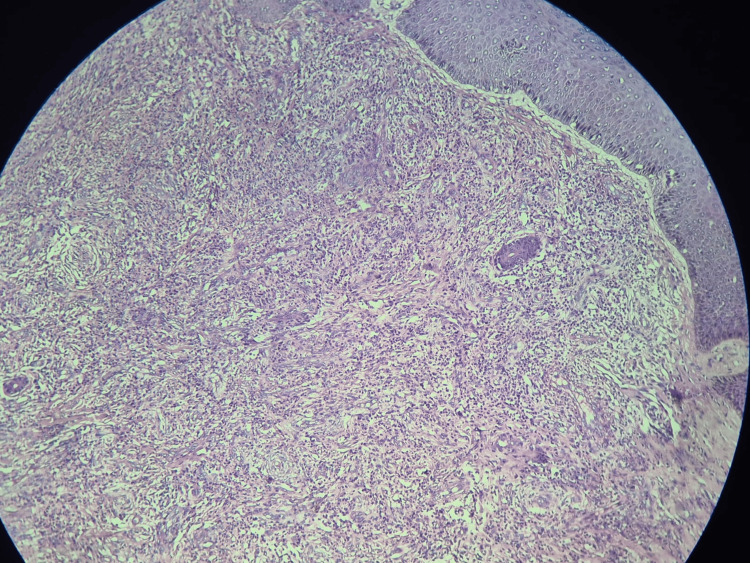
Histopathology result image 1 Microscopy image shows a lesion with a dermal and perivascular vasculopathy reaction pattern.

**Figure 6:**
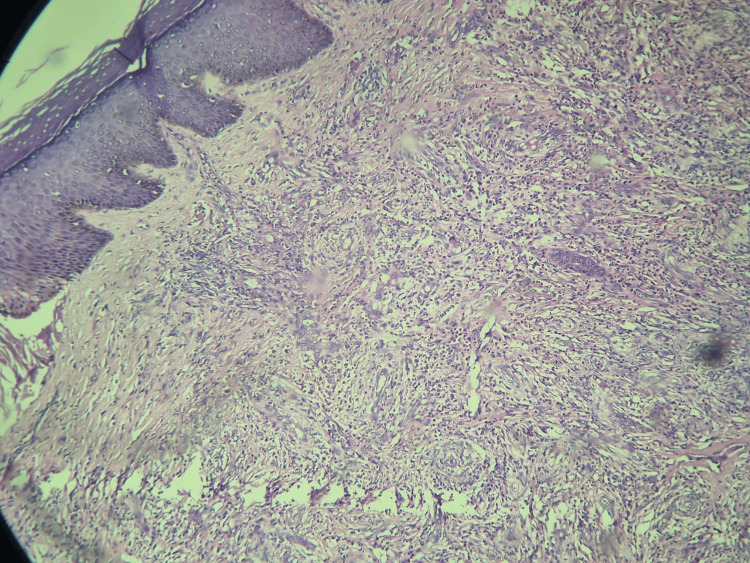
Histopathology result image 2 Microscopy shows dermal perivascular fibrosis in onion skinning pattern with polymorphous infiltrate of predominantly neutrophils, few lymphocytes, macrophages and occasional eosinophils.

**Figure 7:**
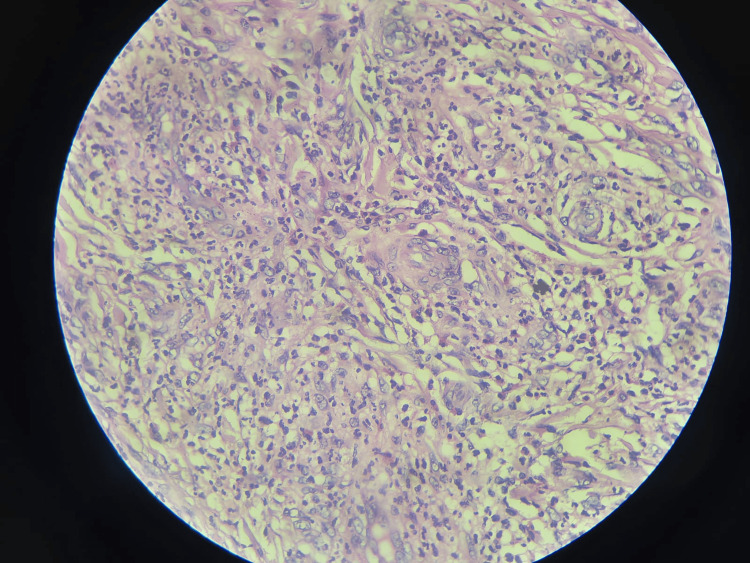
Histopathology result image 3 The image shows classic small vessel vasculitis with focal fibrinoid necrosis.

The new development of pain in the existing lesions required further investigations. The histopathology report confirmed the diagnosis. Histological features were consistent with erythema elevatum diutinum (Figures 5-7).

## Discussion

Erythema elevatum diutinum (EED) is an uncommon, chronic inflammatory, painless skin lesion that typically presents as violaceous, red-brown, yellow papules, nodules, or plaques that are located on the extensor regions of the limbs, particularly the hands, forearms, elbows, feet, and ankles [10]. The lesions may be asymptomatic or present with pain, burning sensations, or pruritus; these may also ulcerate or form crusts. The course in EED can last for months or even years. While the exact cause of EED remains unknown, some cases may be associated with underlying systemic diseases such as lupus, sarcoidosis, or lymphoma, and autoimmune diseases [11,12]. By understanding the classical presentation, healthcare professionals can better recognize EED and differentiate it from other skin conditions. Atypical EED can present with a variety of features that deviate from the classic presentations, making diagnosis challenging. Here’s a breakdown of some difficulties associated with atypical EED: While typically violaceous, red-brown, or yellow, lesions might exhibit unusual colors or a more uniform hue. EED lesions can be smaller or much larger than the usual 2-5 cm range, potentially mimicking other conditions [13]. Although extensor regions of the limbs are the most common sites, atypical presentations involve less common areas like the trunk, buttocks, or head.

This can lead to confusion with other skin diseases [14]. Atypical EED can be surprisingly painful, which is not a typical characteristic. This pain may mimic other inflammatory skin conditions or lead to misdiagnosis as a musculoskeletal issue. Lesions are often scattered; however, atypical presentations may involve clustered lesions, further adding to diagnostic challenges. This atypical presentation can resemble various other skin conditions, such as sarcoidosis, lupus erythematosus, granuloma annulare, or even bacterial infections. This case report presented an atypical manifestation of EED with painful swelling in the lesions. While the exact cause of pain in EED remains elusive [4], several potential mechanisms might explain the patient's pain experience. Increased inflammatory infiltrates composed of immune cells can release various inflammatory mediators that contribute to pain perception [3]. Large or deeply situated lesions might compress nearby nerves, leading to pain and discomfort [13]. Ulceration of the lesions can expose and irritate nerve endings, causing pain and discomfort for the patient [10]. The differential diagnosis for the patient's pain included underlying joint issues (e.g., arthritis), muscular involvement (myositis), and nerve involvement, all of which could coexist with EED [6,8]. Considering the potential inflammatory nature of EED, analgesics and non-steroidal anti-inflammatory drugs were initiated to manage the patient's pain. Treatment for EED typically involves medications such as dapsone [7,10], colchicine, or corticosteroids, antimicrobials, immunomodulatory agents like methotrexate, cyclophosphamide, antibiotics or antifungals, and treatment of underlying systemic disease [2,9]. This case highlights the diagnostic challenges posed by atypical presentations of EED, such as pain. Despite initial medical treatment, the patient's symptoms persisted, necessitating an excisional biopsy with split-thickness skin grafting (SSG) to improve their quality of life.

## Conclusions

Erythema elevatum diutinum is a rare disease in which patients present with skin lesions that are usually painless. In some cases, there are typical symptoms such as pain, which may lead to a diagnostic dilemma. In such scenarios, it is important to reevaluate the patient as a whole and tailor the treatment options for the benefit of the patient. A thorough knowledge of this rare disease and its varied presentations will help in a multimodal approach to the treatment. Finally, the main objective of this report is to bring awareness about the possibility of the occurrence of atypical presentation of EED, such as pain. In the long run, medical treatment of EED might be unsatisfactory and require other treatment modalities. Hence, this patient underscores the importance of close follow-up and interventional procedures.
